# THE EPIDEMIOLOGY OF ALZHEIMER’S DISEASE AND RELATED DEMENTIAS AMONG ARAB AMERICANS

**DOI:** 10.1093/geroni/igz038.1731

**Published:** 2019-11-08

**Authors:** Florence Dallo, Tiffany Kindratt

**Affiliations:** 1 Oakland University, Rochester, Michigan, United States; 2 University of Texas Southwestern Medical Center, Dallas, Texas, United States

## Abstract

In the United States (U.S.), Alzheimer’s Disease and Related Dementias (ADRD) afflict over 4.7 million individuals ages 65 or older. Most studies compare the prevalence of ADRD between minorities and whites. Arab Americans are a subgroup of whites, and ADRD is not understood among Arab Americans. The overall goal of this study is to estimate the prevalence of and risk factors for ADRD among Arab Americans ages 45 or older compared to non-Hispanic whites, non-Hispanic blacks, Hispanics and Asian Americans. Data for 2000-2017 from the National Health Interview Survey (NHIS) using the region of birth question was be used (N=222,219). Percents, chi-square and logistic regression will be estimated. Age- and sex-adjusted prevalence of ADRD was 10.3% for foreign-born Arab Americans compared to approximately 7.5% for US-born non-Hispanic whites (NHW), blacks and Asians. The prevalence of ADRD was 8.6% for Hispanics (all p-values <.0001). When controlling for age and sex, Arab Americans were 1.4 times (OR=1.02,1.93) more likely to have ADRD compared to US-born NHW. This is the first study to focus on ADRD among Arab Americans and the findings suggest ADRD is a burden in this population. Future studies should capture other generations of Arab Americans to better understand the trend of ADRD among this understudied, often invisible population.

